# Engineering of Pentose Transport in *Saccharomyces cerevisiae* for Biotechnological Applications

**DOI:** 10.3389/fbioe.2019.00464

**Published:** 2020-01-29

**Authors:** Jeroen G. Nijland, Arnold J. M. Driessen

**Affiliations:** Molecular Microbiology, Groningen Biomolecular Sciences and Biotechnology, University of Groningen, Groningen, Netherlands

**Keywords:** pentose transport, D-xylose, L-arabinose, yeast, bioethanol

## Abstract

Lignocellulosic biomass yields after hydrolysis, besides the hexose D-glucose, D-xylose, and L-arabinose as main pentose sugars. In second generation bioethanol production utilizing the yeast *Saccharomyces cerevisiae*, it is critical that all three sugars are co-consumed to obtain an economically feasible and robust process. Since *S. cerevisiae* is unable to metabolize pentose sugars, metabolic pathway engineering has been employed to introduce the respective pathways for D-xylose and L-arabinose metabolism. However, *S. cerevisiae* lacks specific pentose transporters, and these sugars enter the cell with low affinity via glucose transporters of the Hxt family. Therefore, in the presence of D-glucose, utilization of D-xylose and L-arabinose is poor as the Hxt transporters prefer D-glucose. To solve this problem, heterologous expression of pentose transporters has been attempted but often with limited success due to poor expression and stability, and/or low turnover. A more successful approach is the engineering of the endogenous Hxt transporter family and evolutionary selection for D-glucose insensitive growth on pentose sugars. This has led to the identification of a critical and conserved asparagine residue in Hxt transporters that, when mutated, reduces the D-glucose affinity while leaving the D-xylose affinity mostly unaltered. Likewise, mutant Gal2 transporter have been selected supporting specific uptake of L-arabinose. In fermentation experiments, the transporter mutants support efficient uptake and consumption of pentose sugars, and even co-consumption of D-xylose and D-glucose when used at industrial concentrations. Further improvements are obtained by interfering with the post-translational inactivation of Hxt transporters at high or low D-glucose concentrations. Transporter engineering solved major limitations in pentose transport in yeast, now allowing for co-consumption of sugars that is limited only by the rates of primary metabolism. This paves the way for a more economical second-generation biofuels production process.

## Introduction

Lignocellulosic biomass, from hardwood, softwood, and agricultural residues, is generally regarded as a promising feedstock for the production of sustainable energy fuels. Also because the readily fermentable agricultural feedstocks like sugar cane and corn interfere in the world food demand (Zaldivar et al., 2002; Solomon, 2010). Lignocellulosic biomass is mainly composed of the carbohydrate polymers cellulose, hemicellulose, and lignin in which the first two are substrates for bioethanol production (Lynd et al., 2005). To release the sugars from the lignocellulosic biomass, hydrolysis is applied which yields a mixture of hexose (mainly from cellulose but also from hemicellulose) and pentose sugars (from hemicellulose). The majority of the hexose and pentose sugars in the hemicellulose fraction is D-glucose and D-xylose, respectively, in a typical mass ratio of 2:1 (Lee, 1997; Carroll and Somerville, 2009; Gírio et al., 2010). However, also L-arabinose (pentose) contributes up to 4.5% of the total amount of carbohydrates in for instance rice straw (Lee, 1997). In industrial fermentation processes, the yeast *Saccharomyces cerevisiae* is generally used for bioethanol production from lignocellulosic biomass. Since *S. cerevisiae* cannot naturally ferment pentose sugars like D-xylose and L-arabinose, specific pentose metabolism pathways have been introduced.

### Pentose Metabolism

**Figure 1:**
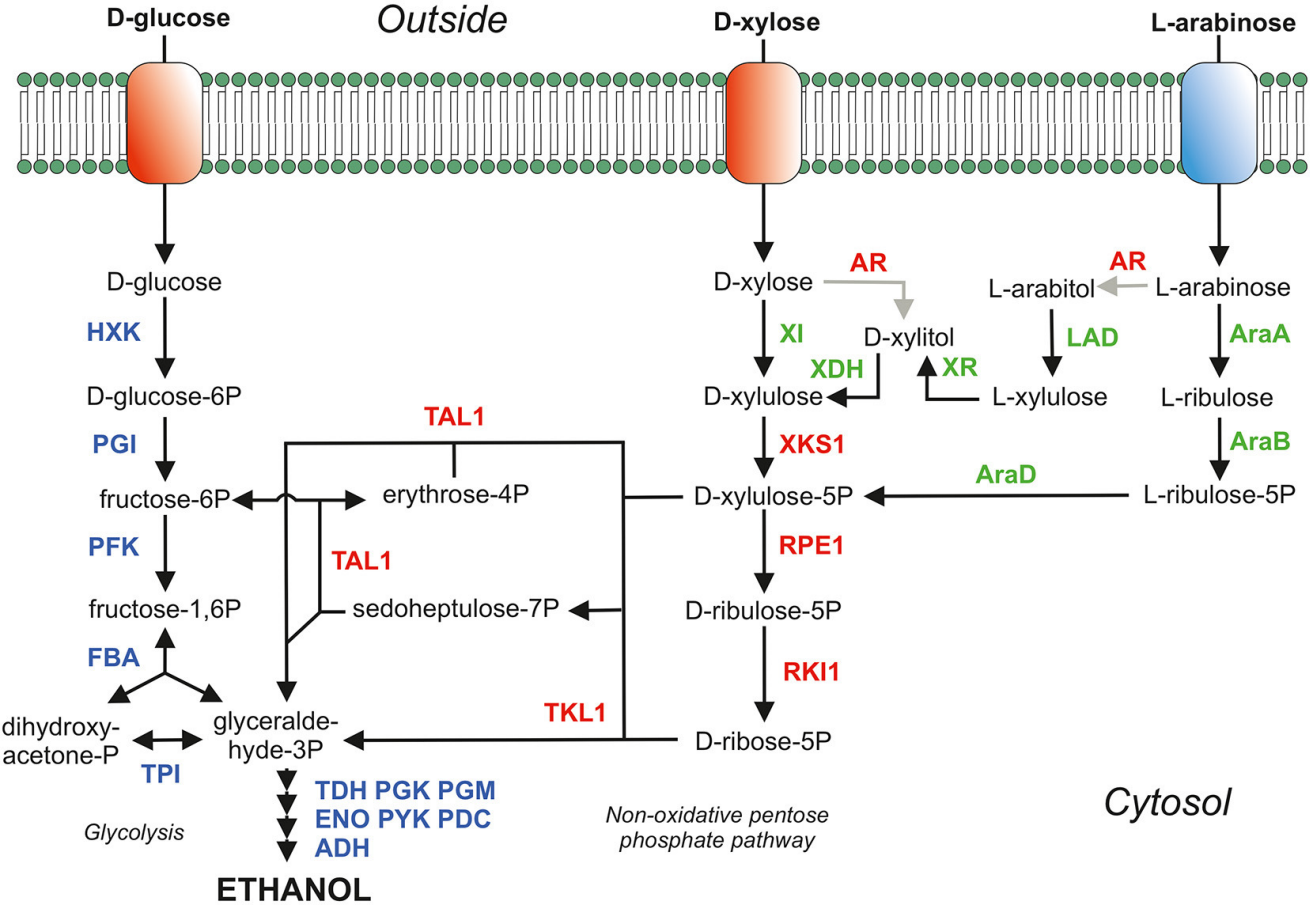
Main sugar consumption pathways in *S. cerevisiae*. The proteins depicted in blue belong the glycolysis and ethanol production (fermentation). Proteins in red are homologous over-expressed proteins of the pentose phosphate pathway (PPP) and AR; the aldose/xylose/arabinose reductase (EC:1.1.1.21) which is over-expressed in the yeast strains expressing XDH (xylitol dehydrogenase; EC:1.1.1.9) but is deleted in yeast strains expressing XI (xylose isomerase; EC:5.3.1.5). In green are depicted the heterologously over-expressed proteins to metabolize D-xylose (XI and XDH) and the proteins for L-arabinose metabolism [AraA (isomerase; EC:5.3.1.4), AraB (ribulokinase; EC:2.7.1.16) and AraD (epimerase; EC:5.1.3.4)]. To prevent arabitol formation in an L-arabinose consuming yeast strain expressing the AraBAD pathway the AR (aldose reductase; EC:1.1.1.21; *gre3* gene) was deleted. The AR was, however, over-expressed in the L-arabinose pathway expressing LAD (L-arabinitol 4-dehydrogenase; EC:1.1.1.12) and XR (L-xylulose reductase; EC:1.1.1.10). Adapted from De Waal P.P., patent WO2003/062430 and WO2008/041840, with permission.

*S. cerevisiae* has been engineered into a D-xylose-fermenting strain via either the introduction of a xylose reductase and xylitol dehydrogenase (Kotter and Ciriacy, 1993; Tantirungkij et al., 1994; Jeffries, 2006; Hahn-Hagerdal et al., 2007; Hou et al., 2009) or a fungal (Kuyper et al., 2003, 2004, 2005b; Hector et al., 2008) or bacterial (Brat et al., 2009; de Figueiredo Vilela et al., 2013; Demeke et al., 2013a,b) xylose isomerase (Figure 1). The xylose reductase and xylitol dehydrogenase pathway both requires co-factors (NADPH and NAD^+^, respectively) which potentially cause an redox imbalance and accumulation of by-products such as xylitol and glycerol. However, re-balancing the intracellular redox cofactor levels or change the cofactor specificities of XR or XDH has significantly improved the D-xylose consumption rate (Verho et al., 2003; Watanabe et al., 2007a,b; Matsushika et al., 2008; Hou et al., 2009, 2014; Zhou et al., 2012). In the xylose reductase and xylitol dehydrogenase pathway as well as in the xylose isomerase pathway, xylulose is phosphorylated to xylulose-5-phosphate, which is further metabolized through the pentose phosphate pathway (PPP). In the PPP, xylulose-5-phospate is converted into fructose-6-phosphate and glyceraldehyde-3-phosphate in a molar ratio of 2:1, and these phosphorylated compounds are subsequently metabolized via glycolysis. Various additional genetic modifications have been applied to both variants of these xylose-fermenting strains e.g., overexpression of the pentose phosphate pathway (Johansson and Hahn-Hägerdal, 2002; Karhumaa et al., 2005, 2006; Kuyper et al., 2005a; Bera et al., 2011), deletion of the *gre3* gene (Traff et al., 2001; Karhumaa et al., 2005, 2006; Kuyper et al., 2005a), deletion of the *pmr1* gene (Verhoeven et al., 2017) and many other genes as reviewed by Moyses et al. (2016).

L-arabinose utilization has been explored significantly less compared to D-xylose utilization due to the lower abundance of L-arabinose in lignocellulosic biomass. Like D-xylose, L-arabinose can be utilized by *S. cerevisiae* via two pathways: an isomerization pathway consisting of L-arabinose isomerase (AraA), L-ribulokinase (AraB), and L-ribulose-5-phosphate 4-epimerase (AraD) from bacteria such as *Escherichia coli, Bacillus subtilis*, and *Lactobacillus plantarum* (Sedlak and Ho, 2001; Becker and Boles, 2003; Wisselink et al., 2007) or a reduction/oxidation-based pathway consisting of an aldose reductase (AR), L-arabinitol 4-dehydrogenase (LAD), L-xylulose reductase (LXR), D-xylulose reductase (XDH), and a xylulokinase (XK) (Richard et al., 2003; Hahn-Hagerdal et al., 2007; Bettiga et al., 2009). L-arabinose metabolism continues, in both variants of L-arabinose metabolism, in the formation of D-xylulose-5-phosphate (Figure 1). Therefore, the PPP genes were also over-expressed yielding increased L-arabinose consumption rates (Becker and Boles, 2003; Hahn-Hagerdal et al., 2007; Wisselink et al., 2007; Bettiga et al., 2009).

### Monosaccharide Transport

In yeast, sugar transport is facilitated by the sugar porter family which is the largest within the major facilitator superfamily (MFS), and includes proteins from bacteria, archaea and eukaryotes, with a high level of structural and functional similarity (Maiden et al., 1987; Baldwin and Henderson, 1989; Henderson and Maiden, 1990). Although the proteins belonging to the MFS family exhibit strong structural conservation, they may share little sequence similarity (Vardy et al., 2004). These permeases consist of two sets of six hydrophobic transmembrane-spanning (TMS) α-helices connected by a hydrophilic loop. In yeast, many monosaccharide transporters operate by facilitated diffusion, an energy-independent mechanism that equilibrates the monosaccharide concentration in the cytoplasm with that of the extracellular medium. In sugar transporters six conserved motifs (K/RXGRR, RX_3_GX_3_G/A, PESPR, PETK, SVQWR, and GRXLXGXGXGX_6_PXYXSEXAPX_3_RG XLX_4_QLXITXGX_3_A) have now been found, irrespective of their mechanism or substrate specificity (Maiden et al., 1987; Henderson and Maiden, 1990; Griffith et al., 1992; Horák, 1997; Pao et al., 1998; Young et al., 2014). D-glucose transport in *S. cerevisiae* is mediated by the hexose transporter (Hxt) family of sugar transporters (Kruckeberg, 1996; Boles and Hollenberg, 1997). These transporters mediate facilitated diffusion of the hexose sugars and are usually of low affinity but high capacity. A strain, in which the main sugar transporter genes *hxt1-17* and *gal2* were deleted, was found to be unable to grow on D-glucose (Wieczorke et al., 1999; Hamacher et al., 2002). A similar deletion strain, lacking only the main expressed hexose transporters (*hxt1-7* and *gal2*) shows minor growth rates on D-glucose (Reifenberger et al., 1995).

*S. cerevisiae* lacks specific D-xylose or L-arabinose transporters, but still is capable of transporting these pentoses via the endogenous Hxt family of D-glucose transporters and the galactose permease, Gal2, respectively (Kou et al., 1970). This transport is of low affinity, and D-xylose transport is readily outcompeted by D-glucose. This implies that cells only start to transport and metabolize D-xylose once D-glucose is depleted which is undesirable for a robust industrial bioethanol process that must rely on the co-consumption of the sugars available in the growth medium. To circumvent the problem of poor D-xylose and L-arabinose transport, various studies have attempted to provide *S. cerevisiae* with a specific pentose transporter from a heterologous source which is discussed in the next section.

## Heterologous Expression of Pentose Transporters

Because *S. cerevisiae* lacks specific pentose transporters, one important strategy in the engineering of pentose metabolism is the introduction of specific pentose transporters from heterologous hosts, i.e., other yeast or fungi, bacterial and transporters of plants. The following section summarizes the main approaches and achievements.

### Heterologous Xylose Transporters

***Ci*Gxf1 and** ***Ci*Gxs1**—Two transporters have been isolated from *Candida intermedia* PYCC 4715: the high-capacity and low-affinity D-glucose/D-xylose facilitated diffusion transporter *Ci*Gxf1 and the high-affinity D-xylose-proton symporter *Ci*Gxs1. Both genes were expressed in *S. cerevisiae* and the kinetic parameters of D-glucose and D-xylose transport were determined (Leandro et al., 2006; Young et al., 2014) (Table 1). *Ci*Gxf1 was expressed in the various recombinant xylose-fermenting *S. cerevisiae* strains in which it displayed approximately a 2-fold lower K_m_ value for D-xylose transport compared to a control strain that relies on the endogenous set of Hxt transporters. In aerobic batch cultivation, the specific growth rate was significantly higher at low D-xylose concentration (4 g/L), when *Ci*Gxf1 was expressed, whereas it remained unchanged at high D-xylose concentration (40 g/L). These results suggest recombinant xylose-utilizing *S. cerevisiae* only benefit from such specific transporters at low D-xylose concentrations (Runquist et al., 2009; Fonseca et al., 2011; Tanino et al., 2012; Diao et al., 2013). Furthermore, *Ci*Gxf1 was used in various *S. cerevisiae* strains and contributed to increased growth and D-xylose consumption rate (Fonseca et al., 2011; Olofsson et al., 2011; Diao et al., 2013).

**Table 1:** Kinetics of endogenous and heterologous expressed xylose transporters in *S. cerevisiae*.

	**D-xylose**		**D-glucose**		
	**K_**m**_ (mM)**	**V_**max**_ (nmol/mgDW.min)**	**K_**m**_ (mM)**	**V_**max**_ (nmol/mgDW.min)**	**Author**
Hxt36	107.9 ± 12.1	62.5 ± 5.9	6.1 ± 0.1	60.2 ± 2	Nijland et al., 2014
Hxt36 N367I	39.8 ± 5.6	23 ± 3	–	±0	Nijland et al., 2014
Hxt36 N367A	24.9 ± 3.4	29.1 ± 0.4	170.7 ± 37.8	70.7 ± 8.4	Nijland et al., 2014
Hxt7	130 ± 9	110 ± 7	nd^a^	nd^a^	Saloheimo et al., 2007
Hxt7	200.3 ± 13.2	67 ± 2	0.5 ± 0.1	26 ± 1.1	Farwick et al., 2014
Hxt7 N370S	169.9 ± 26.3	24.1 ± 1.6	–	±0	Farwick et al., 2014
Hxt7	161.2 ± 22	101.6 ± 6.5	nd^a^	nd^a^	Reider Apel et al., 2016
Hxt7 F79S	228.8 ± 45.9	186.4 ± 20.1	nd^a^	nd^a^	Reider Apel et al., 2016
Gal2	225.6 ± 15.8	91.3 ± 3.2	1.5 ± 0.2	27.2 ± 0.9	Farwick et al., 2014
Gal2 N376F	91.4 ± 8.9	37.3 ± 1.3	–	±0	Farwick et al., 2014
Gal2 N376V	168.3 ± 31.6	28.4 ± 2.3	22.1 ± 1.8	50.5 ± 1.4	Farwick et al., 2014
Hxt11	84.2 ± 10	84.6 ± 2.4	33.4 ± 2.1	156.4 ± 7.6	Shin et al., 2015
Hxt11 N376D	106.7 ± 21.7	86.5 ± 2	87 ± 6.4	197.8 ± 11.4	Shin et al., 2015
Hxt11 N376T	46.7 ± 2.7	76.2 ± 4.8	194.4 ± 47.9	238.6 ± 7.4	Shin et al., 2015
Hxt11 N376M	50.1 ± 9.7	65 ± 6.8	144.9 ± 36	143 ± 17.2	Shin et al., 2015
*Ci*Gxf1	48.7 ± 6.5	10.8 ± 1.0^b^	2 ± 0.6	1.4 ± 0.2^b^	Leandro et al., 2006
*Ci*Gxs1	0.4 ± 0.1	0.39 ± 0.09	0.012 ± 0.004	0.0043 ± 0.0003	Leandro et al., 2006
*Ci*Gxs1	0.026 ± 0.06	7.23 ± 0.6	nd^a^	nd^a^	Young et al., 2014
*Ci*Gxs1 F38I39M40	0.72 ± 0.12	15.01 ± 2.38	±0	±0	Young et al., 2014
*Ci*Gxs1	0.08 ± 0.02	5.68 ± 0.3	nd^a^	nd^a^	Young et al., 2012
*Ci*Gxs1 2.1	1.58 ± 0.49	11.03 ± 3.71	nd^a^	nd^a^	Young et al., 2012
*Ci*Gxs1 2.2	1.2 ± 0.05	3.52 ± 0.27	nd^a^	nd^a^	Young et al., 2012
*Ci*Gxs1 2.3	1.25 ± 0.32	10.91 ± 1.44	nd^a^	nd^a^	Young et al., 2012
*Ps*Sut1	145 ± 1	132 ± 1	1.5 ± 0.1	45 ± 1	Weierstall et al., 1999
*Ps*Sut2	49 ± 1	41 ± 1	1.1 ± 0.1/55 ± 11^c^	3.3 ± 0.1/28 ± 4^c^	Weierstall et al., 1999
*Ps*Sut3	103 ± 3	87 ± 2	0.8 ± 0.1/31 ± 0.1^c^	3.7 ± 0.1/22 ± 0.1^c^	Weierstall et al., 1999
*Ss*Xut3	4.09 ± 1.08	11.31 ± 2.31	nd^a^	nd^a^	Young et al., 2012
*Ss*Xut3 1.1	2.02 ± 0.40	15.67 ± 0.87	nd^a^	nd^a^	Young et al., 2012
*Ss*Xut3 1.2	1.73 ± 0.93	6.65 ± 2.64	nd^a^	nd^a^	Young et al., 2012
*An*HxtB	0.54 ± 0.08	19 ± 1.33	nd^a^	nd^a^	dos Reis et al., 2016

a *Not determined*.

b *Data extracted from figure*.

c *Data fitted more accurately to two transport components*.

*Ci*Gxs1 is the first yeast D-xylose/D-glucose-H^+^ symporter to be characterized at the molecular level (Leandro et al., 2006). Unlike facilitated diffusion, coupling to the proton motive force allows cells to accumulate sugars against their concentration gradient even at low extracellular sugar concentrations. Such conditions can be favorable in order to saturate the intracellular metabolic enzymes such as the xylose isomerase that exhibits a low affinity for its substrates (Lee et al., 2017). Overexpression of *Ci*Gxs1 improved D-xylose consumption and ethanol production in a yeast harboring an XI-based xylose metabolic pathway (Tanino et al., 2012) but transport occurred at low rates, i.e., V_max_ is 5 nmol/mgDW.min. Selection for improved growth on D-xylose yielded transporter mutants with vastly improved V_max_ values (Table 1) and these displayed an increase in high cell density sugar consumption rates. Analysis of the mutations highlighted several important residues influencing transporter function including a point mutation at F40 of *Ci*Gxs1 (Young et al., 2012). Several other mutations (M40V, I117F, and N326H), obtained via error-prone PCR, yielded a *Ci*Gxs1 transporter with substantially improved D-xylose transport rates in the presence of D-glucose and even enabled co-utilization of D-glucose and D-xylose (Li et al., 2016). Young et al. rewired the *Ci*Gxs1 transporter into a more specific xylose transporter via mutagenesis of a conserved motif (G-G/F-X-X-X-G) yielding a mutant (*Ci*Gxs1 V38F L39I F40M) that abrogated the D-glucose uptake and slightly increased the D-xylose uptake rate although D-glucose still inhibited growth on D-xylose (Young et al., 2014).

**At5g59250/At5g17010**—These genes encode sugar transporters from *Arabidopsis thaliana*, and were classified as xylose transporter homologs based on sequence similarity to known xylose–H^+^ symporters (Johnson and Thomas, 2007). These proteins were heterologously expressed in *S. cerevisiae* and found to correctly localize at the cell periphery using GFP fusion proteins. The respective stains showed increased levels of D-xylose accumulation compared to the control strain, but also accumulation of D-glucose was improved (Hector et al., 2008). In a comparative analysis of various heterologously expressed sugar transporters, At5g59250 showed only a slight improvement in the D-xylose uptake kinetics employing a strain harboring the native Hxt landscape, but this result was not statistically significant. In contrast, the aforementioned *Ci*Gxf1 transporter showed the most significant improvement of D-xylose uptake and growth on D-xylose (Runquist et al., 2010).

***Ps*Sut1**, ***Ps*Sut2**, ***Ps*Sut3**, ***Ss*Xut1**, ***Ss*Xut3**, ***Ss*Hxt2.6, and** ***Ss*Qup2**—*Pichia stipitis* (also known as *Scheffersomyces stipitis*) is an excellent xylose-fermenting organism (Does and Bisson, 1989; Grootjen et al., 1991) and is frequently used as a source for heterologous xylose transporters. *Ps*Sut1, *Ps*Sut2, and *Ps*Sut3 encoding glucose transporters were expressed in a *S. cerevisiae hxt* null mutant strain, and all three proteins restored growth on D-glucose. The individual *Ps*Sut proteins show K_m_ values for D-glucose in the millimolar range whereas the affinity for D-xylose was considerably lower. *Ps*Sut2 showed the best affinity for D-xylose (49 mM) but *Ps*Sut1 exhibits a higher V_max_ of 132 nmol/mgDW.min (Table 1) (Weierstall et al., 1999). Furthermore, expression of *Ps*Sut1 in a xylose metabolizing *S. cerevisiae* strain increased both the D-xylose uptake ability and ethanol productivity during D-xylose fermentation. A similar effect was observed for D-glucose using this transporter (Katahira et al., 2008). Improved D-xylose uptake and utilization was demonstrated with a strain that expressed *Ps*Sut1 while still harboring the native Hxt landscape, but this was only observed when cells were grown on D-xylose (Runquist et al., 2010). *Ss*Xut1 and *Ss*Xut3, isolated from *Scheffersomyces (Pichia) stipitis*, exhibit moderate sugar transport rates but with a preference for D-xylose. When expressed in a *S. cerevisiae hxt* null strain these transporters also supported growth on D-glucose (Young et al., 2011). D-xylose uptake via *Ss*Xut1 was confirmed by high density fermentations on solely D-xylose or D-glucose/D-xylose (de Sales et al., 2015). The E538K mutation in *Ss*Xut3 improved both the affinity and V_max_ for D-xylose (see Table 1), and also allowed better growth on low concentrations of D-xylose (Young et al., 2012). In another approach, the *hxt* null strain (Δ*hxt1-7* and Δ*gal2*) of xylose fermenting *S. cerevisiae* strain was transformed with a genomic DNA library from *S. stipitis* and screened for sustaining growth on D-xylose. This led to the identification of three transporter genes, i.e., the previously identified *Ss*Xut1 permease and two new transporter genes, *Ss*HXT2.6 and *Ss*QUP2. High cell density fermentations using D-glucose and D-xylose showed that *Ss*Xut1 consumed the highest amount of D-xylose as compared to *Ss*Hxt2.6 and *Ss*Qup2. Although no direct uptake studies were performed with these transporters, they also transport D-glucose and thus are not selective for D-xylose only (de Sales et al., 2015).

***Mgt*05196**—The transporter *Mgt*05196 was isolated from *Meyerozyma (Pichia) guilliermondii*, an anaerobic xylose metabolizing yeast that is equipped with high D-xylose transport rates. Several key amino acid residues of *Mg*t05196 were analyzed by site-directed mutagenesis for improved D-xylose transport. The F432A and N360S mutations enhanced the D-xylose transport activities of *Mg*t05196. Furthermore, the N360F mutation corresponding to the conserved asparagine 376 in Gal2 (Farwick et al., 2014), rendered transport of D-xylose insensitive to D-glucose inhibition. Although this transporter was expressed heterologously in *S. cerevisiae*, growth rates on D-xylose were comparable, albeit slightly lower, to Gal2 supported growth on D-xylose (Wang et al., 2015). No kinetic data was recorded for *Mg*t05196.

***An*HxtB**—This is a glucose transporter from the filamentous fungus *Aspergillus nidulans* (dos Reis et al., 2013). When expressed in an xylose fermenting *S. cerevisiae hxt*-null strain, *An*HxtB supported growth on D-xylose, but growth was slow in line with the low V_max_ (19 nmol/mgDW.min) of this transporter. *An*HxtB however shows a high affinity for D-xylose, 0.54 mM (Table 1) however its performance on D-glucose was not tested (Colabardini et al., 2014).

***Bs*AraE**—In order to facilitate D-xylose transport and hence increase xylitol production, an arabinose:H^+^ symporter (*Bs*AraE) from the bacterium *Bacillus subtilis* was expressed in the *hxt* null strain of *S. cerevisiae*. This resulted in a 4-fold increase in D-xylose consumption. When *Bs*AraE was overexpressed in a *S. cerevisiae* strain with the full *hxt* transporter landscape, D-xylose consumption and xylitol production was increased considerably. These experiments were carried out under D-glucose limiting conditions, thus the competitive effect of D-glucose was not tested (Kim et al., 2017).

### Heterologous Arabinose Transporters

***Am*Lat1 and** ***Am*Lat2**—These are specific l-arabinose transporters from the fermenting yeast *Ambrosiozyma monospora*. When *Am*Lat1 and *Am*Lat2 were expressed in a *S*. *cerevisiae* mutant in which the main hexose transporters were deleted, l-arabinose transport was observed. These transporters could not restore growth on d-glucose, d-fructose, d-mannose or d-galactose suggesting a high specificity for l-arabinose. At 100 mM l-arabinose, *Am*Lat1 and *Am*Lat2 showed uptake rates of 0.2 and 4 nmol/mgDW.min, respectively (Table 2), which is much lower than the rate of L-arabinose uptake observed in *A. monospora* (Verho et al., 2011) suggesting expression problems. An *Am*Lat1-mCherry fusion transport protein was found to transport L-arabinose with high affinity (*K*_*m*_ ≈ 0.03 mM) (Londesborough et al., 2014).

**Table 2:** Kinetics of endogenous and heterologous expressed L-arabinose transporters in *S. cerevisiae*.

	**L-arabinose**		**D-xylose**		**D-glucose**		
	**K_**m**_ (mM)**	**V_**textsfmax**_ (nmol/min.mgDW)**	**K_**textsfm**_ (mM)**	**V_**textsfmax**_ (nmol/min.mgDW)**	**K_**textsfm**_ (mM)**	**V_**textsfmax**_ (nmol/min.mgDW)**	**Author**
Gal2	371 ± 19	341 ± 7	nd^a^	nd^a^	nd^a^	nd^a^	Knoshaug et al., 2015
Gal2	57 ± 11	2.2 ± 0.26	nd^a^	nd^a^	nd^a^	nd^a^	Subtil and Boles, 2011
Gal2	nd^a^	nd^a^	225 ± 16	91 ± 3.2	1.5 ± 0.2	27.2 ± 0.9	Farwick et al., 2014
Gal2	335 ± 21	75 ± 5	nd^a^	nd^a^	1.9 ± 21	26 ± 1	Verhoeven et al., 2018
Gal2 N376I	117 ± 16	39 ± 3	nd^a^	nd^a^	101 ± 47	32 ± 18	Verhoeven et al., 2018
Gal2 N376S	186 ± 33	64 ± 2	nd^a^	nd^a^	38 ± 1	28 ± 1	Verhoeven et al., 2018
Gal2 N376T	171 ± 17	65 ± 2	nd^a^	nd^a^	57 ± 1	17 ± 4	Verhoeven et al., 2018
Gal2 N376I T89I	103 ± 40	30 ± 2	nd^a^	nd^a^	±0	±0	Verhoeven et al., 2018
*Am*Lat1	nd^a^	0.2 ± 0.015^b^	nd^a^	nd^a^	No growth^c^	No growth^c^	Verho et al., 2011
*Am*Lat1-mCh	0.03	3 x > amLat1	nd^a^	nd^a^	nd^a^	nd^a^	Londesborough et al., 2014
*Am*Lat2	nd^a^	4 ± 0.25^d^	nd^a^	nd^a^	No growth^c^	No growth^c^	Verho et al., 2011
*Km*Axt1	263 ± 57	57 ± 6	27 ± 3	3.8 ± 0.02	No growth^c^, GI^d^	No growth^c^, GI^d^	Knoshaug et al., 2015
*Pg*Axt1	0.13 ± 0.04	18 ± 0.8	65 ± 8	8.7 ± 0.3	no growth^c^, GI^d^	No growth^c^, GI^d^	Knoshaug et al., 2015
*Ss*AraT	3.8 ± 1.7	0.4 ± 1.7	nd^a^	nd^a^	nd^a^	nd^a^	Subtil and Boles, 2011
*At*Stp2	4.5 ± 2.2	0.6 ± 0.08	nd^a^	nd^a^	nd^a^	nd^a^	Subtil and Boles, 2011
*Pc*AraT	0.13 ± 0.03	5.3 ± 0.2	±0	±0	±0	±0	Bracher et al., 2018
*Nc*Lat1	58 ± 4	1945 ± 50	nd^a^	nd^a^	GI^d^	GI^d^	Li et al., 2015
*Mt*Lat1	29 ± 4	172 ± 6	nd^a^	nd^a^	GI^d^	GI^d^	Li et al., 2015

a *Not determined*.

b *Uptake at 100 mM L-arabinose*.

c *No growth observed on D-glucose*.

d *L-arabinose uptake inhibited by D-glucose (GI; D-glucose inhibited)*.

***Km*Axt1 and** ***Pg*Axt1**—These are L-arabinose transporters from *Kluyveromyces marxianus* and *Pichia (Meyerozyma) guilliermondii*, respectively. Both transporters are also capable of transporting D-xylose. The affinity of *Km*Axt1 and *Pg*Axt1 for L-arabinose is 263 and 0.13 mM, respectively. The high affinity L-arabinose transporter *Pg*Axt1 showed 30-fold lower transports rates compared to *Km*Axt1 (Table 2). Both D-glucose and D-xylose significantly inhibited L-arabinose transport by both transporters, which is an unexpected result as growth on D-glucose could not be restored by either of these transporters. *Km*Axt1 showed a better affinity for D-xylose than for L-arabinose, i.e., 27 vs. 263 mM, respectively. *Pg*Axt1 however is highly specific for L-arabinose as only a low affinity was recorded for D-xylose, i.e., 65 mM (Knoshaug et al., 2015).

***Ss*AraT**—This transporter is of the yeast *S. stipitis* was cloned and identified as L-arabinose transporter allowing growth of *S. cerevisiae* at low L-arabinose concentrations. This was confirmed in uptake experiments yielding an affinity of L-arabinose of 3.8 mM but a low V_max_ of 0.4 nmol/mgDW.min (Table 2). *Ss*AraT appears specific as only poor growth was observed with D-glucose while no growth could be detected on D-xylose (Subtil and Boles, 2011).

***At*Stp2**—The sugar transporter *At*Stp2 from *Arabidopsis thaliana* is a H^+^ symporter with a high affinity for D-galactose (Truernit et al., 1999). When expressed in the *S. cerevisiae hxt* null strain, *At*Stp2 proved to be a high affinity L-arabinose transporter (4.5 mM, Table 2) while it barely supported D-glucose uptake. Nevertheless, L-arabinose uptake by *At*Stp2 was strongly impaired by D-glucose, however, the transporter does not support substantial growth in medium containing 2% D-glucose (Subtil and Boles, 2011). *At*Stp2 had been reported to support uptake of D-glucose when expressed in *Schizosaccharomyces pombe* (Truernit et al., 1999). Failure of *At*Stp2 to enable D-glucose uptake in *S. cerevisiae* might therefore be due to a D-glucose-mediated post-transcriptional inhibitory. The combined data suggests that *At*Stp2 is not specific for L-arabinose.

***Nc*Lat1 and** ***Mt*Lat1**—Two novel H^+^-coupled L-arabinose transporters, *Nc*LAT1 from *Neurospora crassa* and *Mt*LAT1 from *Myceliophthora thermophile*, were identified that exhibit 83% identity but appear to be equipped with different substrate specificities. *Nc*LAT1 has a broad substrate specificity whereas *Mt*LAT1 appears more specific for L-arabinose. The K_m_ values of *Nc*LAT1 and *Mt*LAT1 for L-arabinose were 58 and 29 mM, respectively, with V_max_ values of 1945 and 1729 nmol/mgDW.min, respectively. Transport was only partially inhibited by D-glucose. Upon overexpression of *Nc*LAT1 and *Mt*LAT1 in a L-arabinose metabolizing *S. cerevisiae* strain, L-arabinose utilization, growth and ethanol production was improved. Sequence alignment showed that a conserved asparagine, i.e., N376 of Gal2 (Farwick et al., 2014; Verhoeven et al., 2018), has been replaced into phenylalanine in both *Nc*LAT1 and *Mt*LAT1 (Li et al., 2015). This residue is very critical for the specificity of Hxt transporters for D-glucose, and its substitution in the aforementioned transporters might explain why L-arabinose transport is relatively insensitive to D-glucose inhibition which will be discussed in more detail in the engineering section.

***Pc*AraT**—The putative sugar transporter *Pc*AraT (Pc20g01790) of the filamentous fungus *Penicillium chrysogenum*, is upregulated in L-arabinose-limited cultures compared to D-glucose growth. When expressed in a L-arabinose-fermenting *S. cerevisiae* strain in which GAL2 was deleted, growth on L-arabinose could be restored. *Pc*AraT is a H^+^:L-arabinose symporter with high-affinity for L-arabinose (K_m_ = 0.13 mM) while D-xylose and D-glucose are not transported (Bracher et al., 2018).

Despite the many attempts to express heterologous transporters in pentose-utilizing *S. cerevisiae*, this approach did not yield the solution sought for. The general picture that emerged from those studies are problems with low activity and stability of such foreign transporters likely caused by protein misfolding and subsequent degradation by the yeast endogenous protein quality control mechanisms (Steffensen and Pedersen, 2006; Nikko and Pelham, 2009; Subtil and Boles, 2011; Emmerstorfer et al., 2014; Sen et al., 2016). Also, the high specificity equipped with some of these systems often occurs at the expense of catalytic turnover, while it is essential that these transporters can maintain high rates of transport to keep up with sugar metabolism. Another major disadvantage of the heterologously expressed pentose transporters is that (nearly) all are inhibited by D-glucose therefore preventing co-consumption of D-xylose and D-glucose. Therefore, alternative methods have been explored among which utilizing a mutated endogenous transporter landscape as discussed below.

## Engineering of Endogenous Yeast Hexose Transporters

One potential solution to the stability and turnover issue is the engineering of endogenous Hxt transporters for an improved selectivity toward pentose sugars. The main advantages of endogenous transporters is that they are well-expressed and smoothly integrated in the yeast regulatory systems that control the expression and posttranslational degradation under conditions of high and low extracellular D-glucose concentration. Rational engineering of the substrate specificity is complicated by the lack of an atomic structure of these transporters and thus solely relies on structural modeling. Evolutionary engineering of the yeast sugar transporters turned out to be a very effective method to obtain specificity mutants that could be further tailored by directed mutagenesis as discussed in this section.

### D-Xylose Transport

D-xylose transport in *S. cerevisiae* is mediated by proteins of the Hxt family (Hamacher et al., 2002) and more specifically Hxt1 (Nijland et al., 2016), Hxt2 (Saloheimo et al., 2007; Shin et al., 2015), Hxt3/Hxt36 (Nijland et al., 2014), Hxt4, Hxt5, Hxt7 (Saloheimo et al., 2007), Hxt11 (Shin et al., 2015), and Gal2 (Farwick et al., 2014). A major drawback of all of the endogenous Hxt transporters is their low affinity for D-xylose as compared to D-glucose, which results in D-glucose being the preferred substrate for uptake and metabolism in mixed sugar fermentations (Kotter and Ciriacy, 1993; Lagunas, 1993; van Zyl et al., 1999; Subtil and Boles, 2012). Various techniques have been used to increase D-xylose transport in *S. cerevisiae* focusing on the endogenous Hxt transporters. This involved evolutionary engineering, error-prone PCR, gene shuffling, over-expression, and interference in the endogenous regulatory network. In these experimental approaches two different pentose utilizing yeast strains were employed: (1) a multiple hexokinase deletion strain which is unable to phosphorylate D-glucose and thus cannot grow on D-glucose. Such strains can be used to select for improved D-xylose transport in the presence of high concentrations of D-glucose (Farwick et al., 2014; Nijland et al., 2014, 2017; Reznicek et al., 2015; Shin et al., 2015; Li et al., 2016) and (2) a hexose transporter deletion strain in which the main, or even all, Hxt transporters have been deleted. This strain cannot grow on D-xylose because of an inability to transport this pentose. However, it can be used to express specific sugar transporters and for the selection of mutants that show an enhanced growth on D-xylose (Gonçalves et al., 2014; Reznicek et al., 2015; Nijland et al., 2018). Both approaches yielded various mutations in endogenous sugar transporters that either contribute to an improved D-xylose transport or to a reduced sensitivity toward competitive inhibition by D-glucose. Although several mutations were found in a variety of Hxt transporters that contribute to the specificity change, the most important residue identified is a conserved asparagine present in all Hxt transporters and that fulfills a pivotal role in D-glucose recognition. This asparagine at position 366, 367, 370, and 376 in Hxt11 (Shin et al., 2015), Hxt36 (Nijland et al., 2014), Hxt7 and Gal2 (Farwick et al., 2014), respectively, was mutated to different amino acids, all causing a reduced D-glucose affinity and in some cases improving the D-xylose affinity. In Gal2, the N376F mutation completely abolished the uptake of D-glucose while the affinity for D-xylose increased from 225 to 91 mM (Farwick et al., 2014). The N367A mutation in Hxt36 yielded a transporter with a higher affinity for D-xylose (25 mM) but with a lower V_max_ of 29 nmol/mgDW.min as compared to 37 nmol/mgDW.min for the Gal2 N367F mutant. The same error PCR strategy yielded the Gal2 N376Y mutant (Farwick et al., 2014) which, upon the addition of a M435I mutation, showed an improved V_max_ for D-xylose. This improved the growth rate on D-xylose in the presence of a 4-fold excess of D-glucose. The additional benefit of the M435I mutations was, however, not observed in the Gal2 N376F M435I double mutant (Farwick et al., 2016).

**Figure 2:**
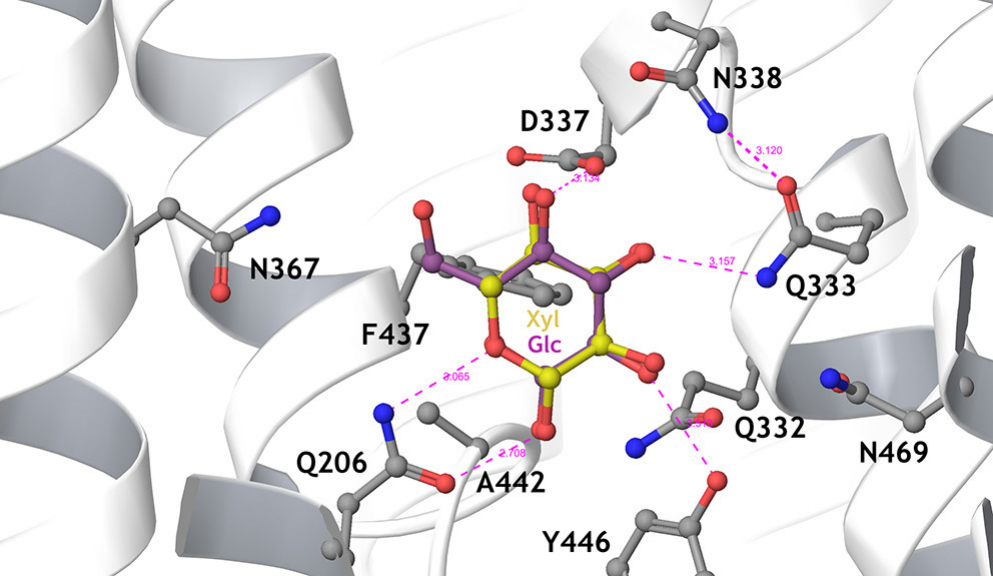
Detailed view of the sugar-binding pocket of the Hxt36 homology model, showing the first shell amino acid side chains that interact with bound glucose (cyan) and xylose (yellow). N367 is located to the left, pointing the side chain toward the 6-OH and 6-CH2 of glucose. Most residues in this pocket are strictly conserved between Hxt36 and XylE, apart from D337 (I in XylE), A442 (G in XylE), Y446 (W in XylE), and N469 (Q in XylE). Reproduced from Nijland et al. (2014) with permission.

In an evolutionary approach using a xylose metabolizing yeast strain lacking the main Hxt transporter, selection for improved growth on D-xylose resulted in the expression of the normally cryptic *hxt11* gene (Shin et al., 2015). Indeed, over-expression of Hxt11 in the transporter-deficient strain resulted in improved growth on D-xylose. Further selection for glucose-insensitive xylose transport employing a quadruple hexokinase deletion yielded mutations at N366 of Hxt11 that reversed the transporter specificity for D-glucose into D-xylose while maintaining high D-xylose transport rates. In particular because of the high xylose transport rates, the Hxt11 N366T mutant enabled the efficient co-fermentation of D-xylose and D-glucose at industrially relevant sugar concentrations when expressed in a strain lacking the *hxt1–7* and *gal2* genes. Among the various Hxt transporters, the Hxt11 N366T mutant is equipped with the most favorable D-xylose uptake characteristics, i.e., a K_m_ of 46 mM and a V_max_ of 76 nmol/mgDW.min (Table 1) (Shin et al., 2015). Another advantage of Hxt11 is that this normally cryptic transporter is stably expressed at high D-glucose concentrations at the plasma membrane without any sign of degradation (Shin et al., 2015, 2017) in contrast to for instance Hxt2 (Kruckeberg et al., 1999; Shin et al., 2017) and Hxt7 (Krampe et al., 1998; Snowdon and van der Merwe, 2012). The structure of Hxt36 (Nijland et al., 2014) (Figure 2) and Gal2 (Farwick et al., 2014) were modeled according to the crystal structure of XylE, a MFS transporter of *Escherichia coli* with D-xylose in the binding site (Sun et al., 2012). Both models clearly show that in the sugar binding site the CH_2_-OH group of D-glucose is in close proximity to N367 or N376, respectively. Any other amino acid that is bulkier or more hydrophobic interfered with the binding of D-glucose. This particular CH_2_-OH group is absent in D-xylose, and thus the mutations leave D-xylose binding mostly unaltered.

Using Gal2 as template, random mutagenesis was used to obtain mutants with an increased affinity for D-xylose. This yielded various mutations but the threonine at position 386 in TMS 8 stands out as the respective mutants allow increased growth rates at low concentrations of D-xylose. This allowed for D-xylose and D-glucose co-consumption at very low sugar concentrations (Reznicek et al., 2015). A similar improvement in D-xylose affinity was observed with mutants of Hxt2 that were obtained via gene shuffling with other Hxt proteins. A mutation of a cysteine to proline at position 505 yielded a 3-fold improvement in D-xylose affinity (Nijland et al., 2018). In another study (Young et al., 2014), mutagenesis of conserved motifs was used to convert Hxt7 into a xylose transporter. These mutations localize to the GGFVFG motif (residues 75-80 and not 36-41 as cited) that is part of TMS 1. The V78I and F79M mutations caused improved growth rates on D-xylose. Like with the other Hxt transporters, mutations at N340 can eliminate D-glucose transport in Hxt7 (Kasahara et al., 2007) and when coupled to the V78I, F79M double mutant, improved growth on D-xylose occurred while growth on D-glucose was defective (Young et al., 2014). In another evolutionary engineering study, a F79S mutation in Hxt7 was obtained that improved D-xylose uptake (Reider Apel et al., 2016).

Remarkably, another mechanism to improve D-xylose metabolism is via the overexpression of Hxt transporters as shown for Hxt1 (Tanino et al., 2012; Gonçalves et al., 2014), Hxt2 and Hxt7 (Gonçalves et al., 2014), with is consistent with the notion that the uptake of D-xylose is the limiting step in metabolism. Although different strain backgrounds were used, in mixed sugar fermentations, over-expression of Hxt1 resulted in fastest sugar consumption (Tanino et al., 2012) while Hxt2 supported the best co-consumption but only at low sugar concentrations (Gonçalves et al., 2014). Evolutionary engineering aimed at improving growth on D-xylose led to the upregulation of Hxt2 (de Vilela et al., 2015). Similar results were obtained via a mutation of the glucose sensitive co-repressor Cyc8 causing the generic upregulation of virtually all *hxt* genes concomitantly with improved D-xylose transport (Nijland et al., 2017). Importantly, all of the abovementioned approaches indeed elevated the D-xylose flux into the cells but, as expected, do not result in sugar co-consumption as D-glucose remains to be the preferred substrate. In a recent study, in which xylitol was produced from lignocellulosic biomass by a recombinant xylose fermenting *S. cerevisiae* strain, improved xylose consumption was achieved by overexpression of the maltose transporter *Sc*Mal11 (Guirimand et al., 2019). Although it was suggested that this phenomenon was due to *Sc*Mal11 supported D-xylose uptake, this was not directly shown in transport assays. Intriguingly, the improved D-xylose consumption was restricted to co-utilization experiments using cellobiose and xylose, and was not observed with a mixture of glucose and xylose.

### L-arabinose Transport

L-arabinose uptake has been studied in less detail compared to D-xylose, likely because of its lesser abundance in lignocellulosic biomass and thus limited economic significance. However, in corn fiber hydrolysates and sugar beet pulp, L-arabinose can account for 26% of the total sugar content (Lynd, 1996; Grohmann and Bothast, 1997). Gal2, the *S. cerevisiae* galactose permease, is capable of transporting arabinose (Kou et al., 1970). Overexpression of Gal2 indeed results in improved L-arabinose uptake (Becker and Boles, 2003; Wang et al., 2013), while a Gal2 deletion strain is unable to metabolize L-arabinose (Wisselink et al., 2009). L-arabinose uptake is inhibited by D-glucose, likely because of competition for transport (Subtil and Boles, 2012). To improve L-arabinose transport, mutations were introduced in Gal2 based on molecular modeling of the substrate binding site. The F85S mutation significantly enhanced the growth rate on L-arabinose and on D-xylose. Furthermore, the F85G mutation increased the L-arabinose transport activity and reduced the specificity of the transporter for D-glucose and D-xylose (Wang et al., 2017). Previously, the corresponding F79S mutation in Hxt7 was reported to improve D-xylose uptake (Reider Apel et al., 2016). This phenylalanine residue is part of the conserved motif “GG/FXXXG” that localizes to TMS1 (Trans membrane spanning domain), and mutations in this region affect the D-xylose and D-glucose transport activity (Young et al., 2014; Knoshaug et al., 2015).

By means of evolutionary engineering of a D-glucose-phosphorylation-negative *S. cerevisiae* strain on mixed sugar feedstocks (glucose-xylose-arabinose), a strain was evolved that grows on L-arabinose in the presence of D-glucose and D-xylose (Verhoeven et al., 2018). Genome sequencing revealed a mutation of the conserved N376 of Gal2 that corresponds to the key residue in Hxt transporters that determines specificity. In Gal2, this residue was mutated into either a serine, threonine or isoleucine. The Gal2 mutants all showed a decreased D-glucose sensitivity of L-arabinose transport which was accompanied by a reduction of the affinity and V_max_ of D-glucose transport (Wisselink et al., 2012; Verhoeven et al., 2018). Reported L-arabinose transport affinities range from 57 mM (Subtil and Boles, 2011) and 371 mM (Knoshaug et al., 2015) (Table 2). Interestingly, in a L-arabinose fermenting *S. cerevisiae* strain that lacks the main Hxt transporters, also Hxt9 and Hxt10 were found to support the uptake of L-arabinose, albeit less efficient than Gal2 (Subtil and Boles, 2011).

## Regulation and Degradation

In *S. cerevisiae*, like in many other yeast species, D-glucose ensures its own efficient metabolism by serving as an environmental stimulus that regulates the quantity, types, and activity of the Hxt transporters, both at the transcriptional and posttranslational level. The Hxt transporters differ in their affinity for D-glucose (Reifenberger et al., 1997), and correspondingly their expression is regulated by different levels of D-glucose (Diderich et al., 1999; Ozcan and Johnston, 1999). Two regulatory pathways exist that involve the transcriptional repressors Mig1 and Rgt1. These pathways interact to ensure that yeast cells expresses the D-glucose transporters best suited at a given concentration of D-glucose in the medium (Nehlin and Ronne, 1990; Ozcan and Johnston, 1995; Kim et al., 2003, 2006; Roy et al., 2013). Furthermore, also degradation of Hxt transporters is tightly linked to the external D-glucose concentration. Hxt1 and Hxt3 are low-affinity D-glucose transporters (Reifenberger et al., 1997), and are expressed at high D-glucose concentrations (Ozcan and Johnston, 1999; Buziol et al., 2008) while they are degraded via ubiquitination at lower D-glucose concentrations (Snowdon and van der Merwe, 2012; Roy et al., 2014; Nijland et al., 2016). Hxt2 and Hxt6/7 are high-affinity D-glucose transporters (Reifenberger et al., 1997) and are expressed when there is no, or only limited amounts of D-glucose present in the medium (Ozcan and Johnston, 1999; Buziol et al., 2008). Concurrently, these transporters are degraded at high D-glucose concentrations (Snowdon and van der Merwe, 2012; Shin et al., 2017). Hxt4 and Hxt5 exhibit an intermediate affinity for D-glucose although Hxt4 is expressed at lower D-glucose concentrations (Ozcan and Johnston, 1999) while Hxt5 is mainly induced when cells are grown on non-fermentable carbon sources and when the growth rate decreases (Verwaal et al., 2002). Little is known about the degradation of Hxt4 but Hxt5 is stably expressed at the membrane at low D-glucose concentrations (Nijland et al., 2016).

Low-affinity D-glucose Hxt transporters all show increased protein degradation at low D-glucose concentrations and are therefore unsuitable to support growth on D-xylose only. Recently it was shown that the degradation of the low-affinity D-glucose transporters Hxt1 and Hxt3 is too fast to enable growth on D-xylose, despite the fact that these transporters support D-xylose transport with high V_max_ values (Nijland et al., 2016). To improve D-xylose and L-arabinose consumption based on these low-affinity D-glucose transporters, protein degradation needs to be prevented, especially at the end of a fermentation when the levels of D-glucose are low. Since protein degradation occurs through ubiquitination of these transporters, the amino-terminal lysine residues of Hxt1, Hxt5 and Hxt36 were mutated into arginine residues (Nijland et al., 2016) or overall protein degradation was reduced in a Rsp5-1ts mutant of the essential E3 ubiquitin ligase, Rsp5 (Snowdon and van der Merwe, 2012). Both studies showed a decreased degradation of the Hxt transporters at low D-glucose concentrations but for only with the respective mutants of Hxt36 (Nijland et al., 2016) improved D-xylose fermentation was observed that could be associated to the improved stability.

## Outlook and Perspective

During the last decade, pentose transport has been extensively studied in the yeast *S. cerevisiae* in order to allow the consumption of the pentose sugars L-arabinose and D-xylose that are present in lignocellulosic biomass. A further goal was to improve co-consumption of hexoses and pentoses. Herein different strategies were investigated: (1) A single Hxt transporter where an endogenous Hxt transporter with improved specificity for D-xylose but that is still able to transport D-glucose, is expressed in a hexose transporter deletion strain (Reznicek et al., 2015; Shin et al., 2015; Farwick et al., 2016; Nijland et al., 2016). Although, co-consumption can be achieved, the total sugar consumption rates remain low, mainly due to D-glucose dependent degradation at either low (e.g., Hxt3) or high (e.g., Hxt7) D-glucose concentrations. (2) Down-regulation of D-glucose consumption via hexokinase mutations yielded an improved co-consumption of D-xylose and D-glucose via an adaptive engineering strategy. This yielded various mutations in D-glucose phosphorylating enzymes (Hxk1, Hxk2, and Glk1). The same phenotype could be obtained by down regulation of the afore mentioned hexokinases which suggests that the rate of intracellular glucose phosphorylation is a decisive factor for metabolic regulations of mixed sugar utilization (Lane et al., 2018). (3) Forced co-consumption in which simultaneous utilization of D-xylose and D-glucose was achieved by deleting the genes encoding glucose-6-phosphate isomerase (Pgi1) and ribulose-5-phosphate epimerase (Rpe1) in a xylose-isomerase-based xylose-fermenting strain. Evolutionary engineering yielded mutations in Hxk2 which improved the rate of the forced co-consumption of D-xylose and D-glucose (Papapetridis et al., 2018). The latter strategy, however, requires stoichiometric mixtures of these two sugars. Apart from these metabolic strategies, the quest for co-consumption was mainly focused on the transport of pentoses which yielded a wealth of information on foreign and endogenous transporters and led to the identification of mutations that either modify the hexose selectivity or transporter stability. The advantage of employing endogenous Hxt transporters is their stable expression and low protein turnover. These transporters are well-integrated in the yeast regulatory network, although protein degradation can be a bottleneck. The disadvantage of these transporters is their low affinity for D-xylose as compared to some of the heterologously expressed xylose transporters, but the latter support D-xylose transport usually with only low uptake rates. Given the wealth of kinetic information available in literature, benchmarking of these transporters based on maximal uptake rates is difficult as this depends on variables such as expression level and the strain, culture and uptake conditions. Through transporter engineering, co-consumption of D-xylose and D-glucose at industrial concentrations has been realized based on single transporter solutions. Although this is a main achievement, to translate this method to real industrial conditions will still be a challenge. The main bottleneck now is that the sugar metabolism flux is distributed over multiple sugars, as the maximal capacity of primary metabolism is exploited. Under such conditions, transport is no longer limiting. Rather primary metabolism represents the main limiting step for faster sugar consumption and shorter fermentation times. Importantly, D-xylose utilization and growth rates are still significantly lower as compared to D-glucose, and thus a careful balance is necessary throughout fermentation. In an industrial setting, obviously robustness remains a main challenge including resistance to toxic byproducts in pretreated feedstocks, and acetic acid that accumulates during fermentation. To make the process industrial ready, a combination of various transporters that will be needed to accommodate the various concentrations of sugar during the entire fermentation and maintain an optimal flux.

## Author Contributions

Both authors designed and conceived the study. JN wrote the initial draft of the manuscript, which was edited and corrected by AD.

### Conflict of Interest

The authors declare that the research was conducted in the absence of any commercial or financial relationships that could be construed as a potential conflict of interest.
